# Development and internal validation of a nomogram for predicting neurobrucellosis in hospitalized patients with brucellosis: a single-center retrospective study

**DOI:** 10.3389/fcimb.2026.1829510

**Published:** 2026-06-29

**Authors:** Qiuyan Chen, Zheng Yin, Yanzi Jin, Fangfang Sun, Yan Wang, Jiaxuan Duan, Xu Wang, Xianrui Xu, Qiang Liu, Ping Yang, Feng Rao, Longnan Wang, Qing Zhang

**Affiliations:** 1 Clinical Medical College, Ningxia Medical University, Yinchuan, Ningxia, China; 2 Department of Neurology, General Hospital of Ningxia Medical University, Ningxia, Key Laboratory of Cerebrocranial Diseases, Incubation Base of National Key Laboratory, Yinchuan, Ningxia, China

**Keywords:** brucellosis, logistic regression, neurobrucellosis, nomogram, risk prediction model

## Abstract

**Background:**

To develop and validate a nomogram based on routinely available clinical indicators for individualized prediction of the risk of neurobrucellosis (NB), thereby providing decision support for early clinical diagnosis and timely treatment while avoiding overtreatment.

**Methods:**

A single-center retrospective study was conducted including 407 patients diagnosed with brucellosis and hospitalized at the General Hospital of Ningxia Medical University between January 1, 2020 and September 1, 2025. Demographic characteristics, comorbidities, clinical symptoms, physical signs, and laboratory findings at the time of first hospitalization were collected. The occurrence of NB served as the outcome variable. Univariate logistic regression analysis was first performed to screen potential predictors, and variables with *P* < 0.05 were subsequently included in a multivariate logistic regression model to identify independent risk factors and construct a nomogram prediction model. The dataset was randomly divided into a training cohort and a validation cohort at a ratio of 7:3 for model development and internal validation. Model discrimination was evaluated using the receiver operating characteristic (ROC) curve and the area under the curve (AUC). Calibration performance was assessed using calibration curves, and clinical utility was evaluated using decision curve analysis (DCA).

**Results:**

A total of 407 patients with brucellosis were included, comprising 275 males (67.6%) and 132 females (32.4%). The incidence of NB was 10.6% (43/407). Compared with non-neurobrucellosis (non-NB) patients, those with NB were younger and had a lower incidence of bone and joint pain. Additionally, NB patients exhibited higher levels of hemoglobin and albumin, but lower levels of fibrinogen, high-sensitivity C-reactive protein, and erythrocyte sedimentation rate (all *P* < 0.05). Univariate logistic regression analysis indicated that age, bone and joint pain, hemoglobin, absolute lymphocyte count (ALC), alanine aminotransferase (ALT), albumin, fibrinogen (FIB), and erythrocyte sedimentation rate (ESR) were associated with the occurrence of NB. Multivariate logistic regression analysis identified bone and joint pain (OR = 0.23, 95% CI: 0.11–0.49), ALC (OR = 1.09, 95% CI: 1.03–1.17), ALT (OR = 0.98, 95% CI: 0.97–0.99), and albumin (OR = 1.13, 95% CI: 1.06–1.20) as independent predictors of NB. The nomogram constructed based on these four variables demonstrated good discrimination and calibration in both the training and validation cohorts. Decision curve analysis showed that the model provided greater net clinical benefit within a reasonable range of threshold probabilities compared with the “treat-all” or “treat-none” strategies.

**Conclusion:**

The nomogram model developed in this study demonstrated good predictive performance and potential clinical applicability. It may serve as a useful tool for early identification of high-risk patients with neurobrucellosis among individuals with brucellosis, thereby facilitating timely and individualized clinical management.

## 1 Introduction

Brucellosis is a zoonotic infectious disease caused by Brucella species. An estimated 1.6-2.1 million new cases are reported globally each year (Laine et al., 2023), and the disease remains highly prevalent in regions with intensive livestock production and relatively limited public health resources. Human infection is mainly acquired through direct contact with infected animals or the consumption of unpasteurized dairy products (About et al., 2023). Occupational exposure, particularly among shepherds, veterinarians, and slaughterhouse workers, is a major risk factor, especially in endemic regions such as the Middle East, Turkey, and parts of China (Jiao et al., 2015; Chen et al., 2023).

NB is a relatively rare but serious complication of brucellosis, accounting for approximately 2%-10% of all brucellosis cases (Buzgan et al., 2010; Maji et al., 2020). NB can involve both the central and peripheral nervous systems and is characterized by marked clinical heterogeneity. Common manifestations include chronic or subacute meningitis, meningoencephalitis, myelitis, cerebrovascular events, cranial nerve involvement, and peripheral neuropathy (Soares et al., 2023). Owing to its nonspecific clinical presentation, as well as the diversity of neuroimaging findings and cerebrospinal fluid abnormalities, neurobrucellosis is easily misdiagnosed in clinical practice as tuberculous meningitis, autoimmune encephalitis, or other infectious diseases of the nervous system. Such misdiagnosis may delay appropriate treatment and even result in irreversible neurological impairment.

At present, the diagnosis of NB mainly relies on a comprehensive assessment of epidemiological history, clinical manifestations, and the results of serological and cerebrospinal fluid examinations. However, in patients at an early stage of the disease or those with atypical clinical presentations, these approaches have substantial limitations in both sensitivity and specificity, which may lead to missed diagnosis, misdiagnosis, or overtreatment. Existing studies on NB have largely focused on retrospective case series or descriptive analyses (Yang et al., 2024; Arazi et al., 2025; Pan et al., 2025), while studies on the development and validation of systematic risk prediction models remain limited, making it difficult to meet the clinical need for early identification and precise decision-making. Therefore, this study aims to identify independent risk factors for NB based on clinical manifestations and serological indicators, and to develop and validate a risk prediction model for neurobrucellosis. The goal is to provide objective and quantitative decision support for the early diagnosis, risk stratification, and individualized treatment of NB, thereby improving the early detection rate while reasonably avoiding unnecessary examinations and treatment.

## 2 Materials and methods

### 2.1 Study population and data sources

This single-center retrospective study consecutively included 407 hospitalized patients with a confirmed diagnosis of brucellosis at the General Hospital of Ningxia Medical University between January 1, 2020 and September 1, 2025, as identified through the electronic medical record system. Baseline demographic data at first hospitalization, including age, sex, ethnicity, occupation, and exposure history, were collected. Information on comorbidities, such as hypertension, diabetes mellitus, and coronary heart disease, was also obtained. Blood pressure on admission was recorded and classified according to the measurements obtained at first hospitalization. Hypotension was defined as systolic blood pressure <90 mmHg or diastolic blood pressure <60 mmHg. Hypertension was defined as systolic blood pressure ≥130 mmHg or diastolic blood pressure ≥80 mmHg. Normal blood pressure was defined as systolic blood pressure of 90–129 mmHg and diastolic blood pressure of 60–79 mmHg. Clinical manifestations and signs, including fever, fatigue, bone and joint pain, night sweats, hepatosplenomegaly, and lymphadenopathy, were recorded. In this study, “bone and joint pain” referred only to nonspecific bone and/or joint pain and did not include confirmed focal musculoskeletal involvement. Laboratory parameters included routine blood test indices (WBC, HGB, PLT, ANC, and ALC), inflammatory and coagulation markers (hs-CRP, ESR, PCT, D-dimer, and fibrinogen), liver function and cell injury markers (ALT, AST, and LDH), renal function indicators (serum creatinine, blood urea nitrogen, and estimated glomerular filtration rate), indicators related to nutritional and protein metabolism status (albumin and globulin), as well as serum ferritin and IL-6.For patients diagnosed with neurobrucellosis, additional data were collected, including neurological manifestations and signs, cerebrospinal fluid parameters, neuroimaging findings, and initial antimicrobial treatment regimens. All data were extracted and verified by uniformly trained investigators using a predesigned standardized case report form. This study was approved by the Medical Ethics Committee of the General Hospital of Ningxia Medical University (Approval No. KYLL-2026-0155) and was conducted in accordance with the Declaration of Helsinki. Given the retrospective nature of the study, the requirement for informed consent was waived by the ethics committee.

Brucellosis was diagnosed when at least one of the following criteria was met (Zheng et al., 2018):(1) Brucella was isolated from blood, other body fluids, or tissue samples, or its nucleic acid was detected;(2) patients presented with typical clinical manifestations of brucellosis and had a positive Rose Bengal plate agglutination test (RBPT) or a positive serum agglutination test (SAT ≥1:100).NB was diagnosed in patients with brucellosis when at least one of the following criteria was fulfilled (Naderi et al., 2022):(1) symptoms and signs suggestive of NB, such as severe and persistent headache interfering with normal daily activities, insomnia, confusion, depression, behavioral changes, urinary incontinence, neck stiffness, or any neurological findings on examination;(2) isolation of Brucella from cerebrospinal fluid (CSF) and/or positive anti-Brucella antibodies in CSF;(3) CSF abnormalities characterized by lymphocytic pleocytosis, elevated protein levels, and decreased glucose levels; or(4) cranial MRI or CT findings suggestive of encephalitis or meningitis.

### 2.2 Inclusion and exclusion criteria

The inclusion criteria were as follows:(1) patients who met the diagnostic criteria for brucellosis;(2) patients who received standardized treatment regularly for at least 1 month; and(3) patients with complete clinical and laboratory data.

The exclusion criteria were as follows:(1) age ≤18 years; (2) concomitant tuberculosis, typhoid fever, paratyphoid fever, rheumatic fever, other natural focal diseases, or confirmed immunodeficiency;(3) concomitant diseases that could interfere with the evaluation of nervous system involvement, such as other infectious or immune-related spondylitis;(4) long-term use of glucocorticoids or immunosuppressive agents, and women during pregnancy or lactation; and(5) cases with missing key information that prevented assessment of outcomes or exposures.

### 2.3 Variable assignment and sample size

The primary outcome was the occurrence of NB. Candidate predictors were prespecified based on published literature and clinical experience, including demographic characteristics (age, sex, ethnicity, occupation, and exposure history), comorbidities (hypertension, diabetes mellitus, and coronary heart disease), clinical manifestations (fever, fatigue, bone and joint pain, night sweats, hepatosplenomegaly, and lymphadenopathy), and laboratory parameters, including routine blood indices, inflammatory and coagulation markers, liver and kidney function markers and indicators related to nutritional status, protein metabolism, and iron metabolism. Sample size was evaluated according to the principle that the number of events per variable (EPV) should be no less than 10. A total of 407 patients with brucellosis were included during the study period, of whom 43 had NB. Since 4 predictors were retained in the final nomogram, the EPV exceeded 10, indicating that the sample size was adequate for multivariable logistic regression analysis.

### 2.4 Statistical analysis

All statistical analyses and figure generation were performed using R software version 4.4.3, and the final figure layout was further refined with the assistance of Adobe Illustrator. Before statistical analysis, data completeness was assessed for all variables required for outcome assessment and model construction. Cases with missing key clinical or laboratory information were excluded during patient selection.

Appropriate statistical methods were selected according to variable type and distribution characteristics. Categorical variables are presented as numbers and percentages [n (%)], and comparisons between groups were performed using the chi-square test or Fisher’s exact test, as appropriate. Continuous variables with a normal distribution are expressed as mean ± standard deviation (SD), and comparisons between groups were conducted using the independent-samples t test. Continuous variables with a non-normal distribution are presented as median and interquartile range [M (Q1, Q3)], and comparisons between groups were performed using the Mann–Whitney U test. The occurrence of NB was used as the dependent variable. Univariate logistic regression analysis was first performed for all candidate predictors, and variables with P < 0.05 were entered into the multivariable logistic regression model. For continuous variables included in the logistic regression models, including age, ALC, ALT, and albumin, the linearity assumption with respect to the logit was assessed using the Box–Tidwell test. No significant deviation from linearity was observed. Independent predictors were identified using stepwise regression, and effect sizes are reported as odds ratios with 95% confidence intervals (95% CIs).Subsequently, the entire study population was randomly divided into a training cohort and an internal validation cohort at a ratio of 7:3 using the caret package in R, with the random seed set to 123 to ensure reproducibility. The final prediction model was developed using the training cohort, and a nomogram was constructed based on the rms package.

Model performance was evaluated from three aspects: (1) discrimination, in which receiver operating characteristic (ROC) curves were plotted and the area under the curve (AUC) was calculated to assess the model’s ability to distinguish between patients with and without NB; (2) calibration, in which calibration curves for the training set were generated using bootstrap resampling (B = 1000), while in the validation set, locally estimated scatterplot smoothing (LOESS) was used to plot the relationship between predicted probabilities and observed event rates; and (3) clinical net benefit, in which decision curve analysis (DCA) was performed to compare the net benefit of three strategies across different threshold probabilities: model-based decision-making, treatment of all patients, and treatment of no patients. All statistical tests were two-sided, and a *P* value < 0.05 was considered statistically significant.

## 3 Results

### 3.1 Baseline characteristics of the patients

A total of 407 hospitalized patients with brucellosis were included, comprising 364 patients without neurobrucellosis (non-NB) and 43 patients with NB, yielding an NB incidence of 10.6% (43/407). In the overall cohort, 275 patients were male (275/407, 67.6%) and 132 were female (132/407, 32.4%).Compared with the non-NB group, patients in the NB group were younger [45.00 (34.50, 55.50) years vs. 53.00 (40.00, 60.00) years, *P* = 0.010], and had a significantly lower incidence of bone and joint pain (30.23% vs. 56.59%, *P* = 0.001). Regarding laboratory parameters, the NB group had significantly higher hemoglobin (HGB) and albumin (ALB) levels than the non-NB group [HGB: 142.00 (128.50, 148.50) g/L vs. 128.00 (114.00, 142.25) g/L, P = 0.002; ALB: 40.40 (36.67, 44.55) g/L vs. 36.08 (32.40, 39.30) g/L, *P* < 0.001]. In contrast, fibrinogen levels were lower in the NB group [2.90 (2.55, 3.69) g/L vs. 3.69 (2.82, 4.33) g/L, *P* = 0.006], and both hs-CRP and ESR levels were also lower (both *P* < 0.05). No statistically significant differences were observed between the two groups in sex, ethnicity, occupation, exposure history, or most other laboratory parameters (*P* > 0.05) (**Table 1**).

**Table 1:** Basic characteristics and differential analysis.

Variables	Total (n = 407)	Non-NB (n = 364)	NB (n = 43)	P
Age	52.00 (39.00, 60.00)	53.00 (40.00, 60.00)	45.00 (34.50, 55.50)	**0.010**
Sex				0.745
Male	275 (67.57)	245 (67.31)	30 (69.77)	
Female	132 (32.43)	119 (32.69)	13 (30.23)	
Ethnicity				0.065
Han	269 (66.09)	246 (67.58)	23 (53.49)	
Non-Han	138 (33.91)	118 (32.42)	20 (46.51)	
Occupation				0.105
Farmer	199 (48.89)	183 (50.27)	16 (37.21)	
Non-Farmer	208 (51.11)	181 (49.73)	27 (62.79)	
Exposure history	221 (54.30)	202 (55.49)	19 (44.19)	0.159
Blood pressure classification				0.543
Low	7 (1.72)	7 (1.92)	0 (0.00)	
Normal	341 (83.78)	303 (83.24)	38 (88.37)	
Hypertension	59 (14.50)	54 (14.84)	5 (11.63)	
Diabetes	25 (6.14)	22 (6.04)	3 (6.98)	1.000
Coronary heart Disease	17 (4.18)	16 (4.40)	1 (2.33)	0.811
Fever	299 (73.46)	266 (73.08)	33 (76.74)	0.606
Bone and joint pain	219 (53.81)	206 (56.59)	13 (30.23)	**0.001**
Fatigue	322 (79.12)	286 (78.57)	36 (83.72)	0.432
Hyperhidrosis	171 (42.01)	158 (43.41)	13 (30.23)	0.098
Hepatomegaly	22 (5.41)	22 (6.04)	0 (0.00)	0.193
Splenomegaly	78 (19.16)	74 (20.33)	4 (9.30)	0.082
Lymphadenopathy	40 (9.83)	38 (10.44)	2 (4.65)	0.350
WBC	5.38 (4.01, 6.97)	5.26 (3.98, 6.93)	5.86 (4.75, 7.59)	0.125
HGB	130.00 (116.00, 143.00)	128.00 (114.00, 142.25)	142.00 (128.50, 148.50)	**0.002**
PLT	210.00 (146.50, 267.00)	210.00 (145.75, 270.25)	227.00 (154.50, 248.50)	0.848
ANC	3.01 (2.02, 4.46)	2.94 (2.00, 4.46)	3.30 (2.23, 4.99)	0.281
ALC	1.72 (1.23, 2.28)	1.69 (1.23, 2.21)	1.80 (1.35, 2.46)	0.194
PLR	117.99 (77.84, 170.42)	117.06 (77.42, 172.03)	121.43 (84.93, 156.65)	0.857
NLR	1.86 (1.10, 2.80)	1.82 (1.10, 2.75)	2.10 (1.19, 3.45)	0.563
ALT	36.70 (20.60, 65.70)	37.45 (20.95, 68.78)	32.20 (17.40, 45.30)	0.097
AST	34.00 (21.00, 57.10)	34.75 (21.00, 60.15)	33.50 (17.65, 50.00)	0.214
LDH	236.00 (180.50, 382.00)	235.00 (181.25, 382.00)	242.00 (178.50, 366.00)	0.985
eGFR	125.47 ± 36.79	124.85 ± 37.27	133.54 ± 30.34	0.474
Creatinine	50.58 (50.58, 55.00)	50.58 (50.58, 55.55)	50.58 (50.58, 50.59)	0.802
Blood Urea Nitrogen	4.92 (4.21, 4.92)	4.92 (4.22, 4.92)	4.92 (4.11, 4.92)	0.645
Albumin	36.50 (32.80, 39.90)	36.08 (32.40, 39.30)	40.40 (36.67, 44.55)	**<.001**
Globulin	30.20 (27.00, 33.25)	30.20 (27.00, 33.30)	30.20 (27.40, 31.75)	0.948
D-Dimer	1.07 (0.64, 2.24)	1.08 (0.66, 2.34)	0.82 (0.47, 2.08)	0.360
FIB	3.59 (2.70, 4.33)	3.69 (2.82, 4.33)	2.90 (2.55, 3.69)	**0.006**
PCT	0.14 (0.07, 0.26)	0.15 (0.07, 0.26)	0.12 (0.07, 0.28)	0.545
Hs-Crp	27.40 (9.85, 49.25)	28.70 (10.00, 49.78)	14.30 (6.00, 40.30)	**0.049**
ESR	18.00 (7.00, 35.50)	18.00 (7.00, 36.00)	11.00 (6.00, 25.00)	**0.036**
Serum Ferritin	406.00 (209.00, 804.70)	412.00 (218.00, 804.70)	313.00 (146.00, 511.50)	0.073
IL-6	16.97 (7.41, 36.70)	17.55 (7.73, 36.90)	10.90 (4.30, 30.57)	0.065

WBC, white blood cell; HGB, hemoglobin; PLT, platelet; ANC, absolute neutrophil count; ALC, absolute lymphocyte count; PLR, platelet-to-lymphocyte ratio; NLR, neutrophil-to-lymphocyte ratio; ALT, alanine aminotransferase; AST, aspartate aminotransferase; LDH, lactate dehydrogenase; eGFR:estimated glomerular filtration rate;D-dimer, D-dimer; FIB, fibrinogen; PCT, procalcitonin; Hs-CRP, high-sensitivity C-reactive protein; ESR, erythrocyte sedimentation rate; IL-6, interleukin-6. Z: Mann-Whitney test, χ²: Chi-square test.

Bold values indicate statistically significant differences.

### 3.2 Clinical manifestations, cerebrospinal fluid findings, and neuroimaging characteristics in patients with neurological brucellosis

The clinical classifications, manifestations, cerebrospinal fluid parameters, and imaging characteristics of 43 patients with neurological brucellosis are as follows: In terms of clinical classification, 22 cases (51.2%) presented with meningitis, 14 cases (32.6%) with meningoencephalitis, 4 cases (9.3%) with myelitis, and 3 cases (6.9%) with peripheral neuropathy; Regarding clinical manifestations, 28 patients (65.1%) primarily presented with headache, 5 (11.6%) with limb weakness, 4 (9.3%) with psychiatric and behavioral abnormalities, 3 (7.0%) with agitation, 2 (4.7%) with limb numbness, and 1 (2.3%) with blurred vision; Cerebrospinal fluid (CSF) analysis revealed typical inflammatory changes in most patients. Thirty-nine patients (90.7%) exhibited elevated CSF protein levels and lymphocytosis, while 26 patients (60.5%) had decreased CSF glucose levels. However, the positivity rate for Brucella culture in CSF was low, with only 5 of 43 patients testing positive, accounting for 11.6%. Neuroimaging abnormalities consistent with neurobrucellosis were identified in only 4 patients, representing 9.3% of the study population (see **Table 2**).

**Table 2:** Clinical manifestations, cerebrospinal fluid findings, and neuroimaging characteristics in patients with neurological brucellosis.

Characteristics	Overall (N = 43)
Clinical type, n (%)	
Meningitis	22 (51.2)
Meningoencephalitis	14 (32.6)
Myelitis	4 (9.3)
Peripheral neuropathy	3 (6.9)
Clinical manifestation, n (%)	
Headache	28 (65.1)
Agitation	3 (7.0)
Blurred vision	1 (2.3)
Limb weakness	5 (11.6)
Limb numbness	2 (4.7)
Psychiatric/behavioral abnormality	4 (9.3)
CSF culture, n (%)	5 (11.6)
CSF protein elevation, n (%)	39 (90.7)
CSF glucose decrease, n (%)	26 (60.5)
CSF Lymphocytosis, n (%)	39 (90.7)
Specific imaging findings of neurobrucellosis, n (%)	4 (9.3)

CSF, cerebrospinal fluid.

### 3.3 Initial treatment regimens of the 43 patients with neurobrucellosis

Among the 43 patients with neurobrucellosis, 36 patients, accounting for 83.7%, received a combination regimen consisting of ceftriaxone, rifampicin, and doxycycline. Ceftriaxone was administered intravenously at a dose of 2 g every 12 hours, rifampicin was administered orally at 600 mg per day, and doxycycline was administered orally at 100 mg twice daily. The remaining 7 patients, accounting for 16.3%, received rifampicin combined with doxycycline. Rifampicin was administered orally at 600 mg per day, and doxycycline was administered orally at 100 mg twice daily. The treatment duration was individualized according to clinical symptoms and cerebrospinal fluid findings.

### 3.4 Logistic regression analysis

Univariate logistic regression analysis identified age, bone and joint pain, HGB, ALC, ALT, albumin, FIB, and ESR as significant factors associated with NB. Age [OR = 0.97, 95% CI: 0.95-0.99, *P* = 0.010], bone and joint pain [OR = 0.33, 95% CI: 0.17-0.66, *P* = 0.002], ALT [OR = 0.99, 95% CI: 0.98-0.99, *P* = 0.033], FIB [OR = 0.73, 95% CI: 0.55-0.98, *P* = 0.036], and ESR [OR = 0.98, 95% CI: 0.96-0.99, *P* = 0.033] were inversely associated with the risk of NB, whereas HGB [OR = 1.02, 95% CI: 1.01-1.04, *P* = 0.008], ALC [OR = 1.07, 95% CI: 1.01-1.13, *P* = 0.022], and albumin [OR = 1.14, 95% CI: 1.07-1.22, *P* < 0.001] were positively associated with NB risk.

**Figure 1:**
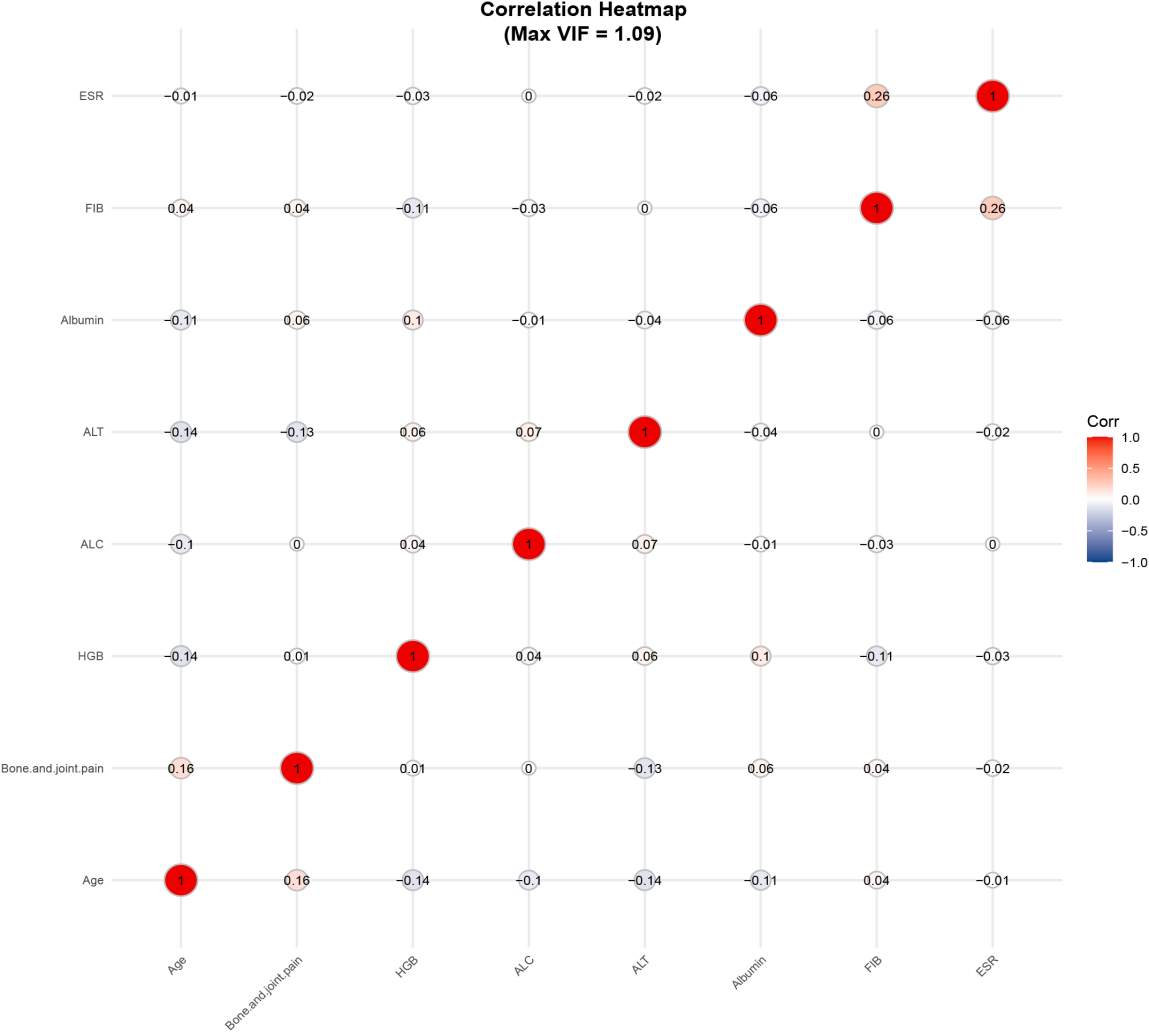
Correlation heatmap for assessing multicollinearity.

The correlation heatmap (**Figure 1A**) demonstrated generally weak correlations among the candidate predictors, with no obvious collinearity. The maximum variance inflation factor (VIF) was 1.09, indicating satisfactory independence among variables and supporting their inclusion in the multivariable regression model. After adjustment in the multivariable logistic regression model, bone and joint pain, ALC, ALT, and albumin remained independently associated with NB. Bone and joint pain [OR = 0.23, 95% CI: 0.11-0.49, *P* < 0.001] and ALT [OR = 0.98, 95% CI: 0.97-0.99, *P* = 0.013] were negatively associated with NB, whereas ALC [OR = 1.09, 95% CI: 1.03-1.17, *P* = 0.006] and albumin [OR = 1.13, 95% CI: 1.06-1.20, *P* < 0.001] were positively associated with NB. Age, HGB, FIB, and ESR lost statistical significance after adjustment for other covariates (*P* > 0.05) (**Table 3**).

**Table 3:** Univariate and multivariate logistic regression analyses.

Variables	Single-factor			Multifactor		
	β	P	OR (95%CI)	β	P	OR (95%CI)
Age	-0.03	0.010	0.97 (0.95 ~ 0.99)	-0.01	0.473	0.99 (0.97 ~ 1.02)
Sex				/	/	/
Male			1.00 (Reference)			
Female	-0.11	0.745	0.89 (0.45 ~ 1.77)	/	/	/
Ethnicity						
Han			1.00 (Reference)			
Non-Han	0.59	0.068	1.81 (0.96 ~ 3.43)	/	/	/
Exposure history	-0.45	0.162	0.63 (0.34 ~ 1.20)	/	/	/
Occupation						
Farmer			1.00 (Reference)			
Non-farmer	0.53	0.108	1.71 (0.89 ~ 3.27)	/	/	/
Hypertension	-0.28	0.573	0.76 (0.28 ~ 2.00)	/	/	/
Diabetes	0.15	0.810	1.17 (0.33 ~ 4.07)	/	/	/
Coronary Heart Disease	-0.66	0.528	0.52 (0.07 ~ 4.00)	/	/	/
Fever	0.20	0.607	1.22 (0.58 ~ 2.56)	/	/	/
Bone and joint pain	-1.10	0.002	0.33 (0.17 ~ 0.66)	-1.47	<.001	0.23 (0.11 ~ 0.49)
Fatigue	0.34	0.434	1.40 (0.60 ~ 3.27)	/	/	/
Hyperhidrosis	-0.57	0.101	0.56 (0.29 ~ 1.12)	/	/	/
Hepatomegaly	-15.49	0.985	0.00 (0.00 ~ Inf)	/	/	/
Splenomegaly	-0.91	0.092	0.40 (0.14 ~ 1.16)	/	/	/
Lymphadenopathy	-0.87	0.242	0.42 (0.10 ~ 1.80)	/	/	/
WBC	0.03	0.603	1.03 (0.93 ~ 1.13)	/	/	/
HGB	0.02	0.008	1.02 (1.01 ~ 1.04)	0.02	0.078	1.02 (1.00 ~ 1.03)
PLT	0.00	0.968	1.00 (1.00 ~ 1.00)	/	/	/
ANC	-0.01	0.734	0.99 (0.92 ~ 1.06)	/	/	/
ALC	0.07	0.022	1.07 (1.01 ~ 1.13)	0.09	0.006	1.09 (1.03 ~ 1.17)
PLR	-0.00	0.987	1.00 (1.00 ~ 1.00)	/	/	/
NLR	-0.01	0.915	0.99 (0.91 ~ 1.09)	/	/	/
ALT	-0.01	0.033	0.99 (0.98 ~ 0.99)	-0.02	0.013	0.98 (0.97 ~ 0.99)
AST	-0.01	0.212	0.99 (0.99 ~ 1.00)	/	/	/
LDH	-0.00	0.699	1.00 (1.00 ~ 1.00)	/	/	/
eGFR	-0.001	0.925	0.99 (0.99 ~ 1.01)	/	/	/
Creatinine	-0.013	0.385	0.99(0.96 ~ 1.02)	/	/	/
Blood Urea Nitrogen	-0.120	0.302	0.89 (0.71 ~ 1.11)	/	/	/
Albumin	0.13	<.001	1.14 (1.07 ~ 1.22)	0.12	<.001	1.13 (1.06 ~ 1.20)
Globulin	0.01	0.503	1.01 (0.97 ~ 1.06)	/	/	/
D-Dimer	0.00	0.997	1.00 (0.96 ~ 1.04)	/	/	/
FIB	-0.31	0.036	0.73 (0.55 ~ 0.98)	-0.18	0.338	0.84 (0.58 ~ 1.21)
PCT	-0.02	0.910	0.98 (0.66 ~ 1.44)	/	/	/
Hs-Crp	-0.01	0.084	0.99 (0.98 ~ 1.00)	/	/	/
ESR	-0.02	0.033	0.98 (0.96 ~ 0.99)	-0.01	0.428	0.99 (0.97 ~ 1.01)
Serum Ferritin	-0.00	0.123	1.00 (1.00 ~ 1.00)	/	/	/
IL-6	-0.01	0.267	0.99 (0.98 ~ 1.01)	/	/	/

### 3.5 Construction and validation of the prediction model

**Figure 2:**
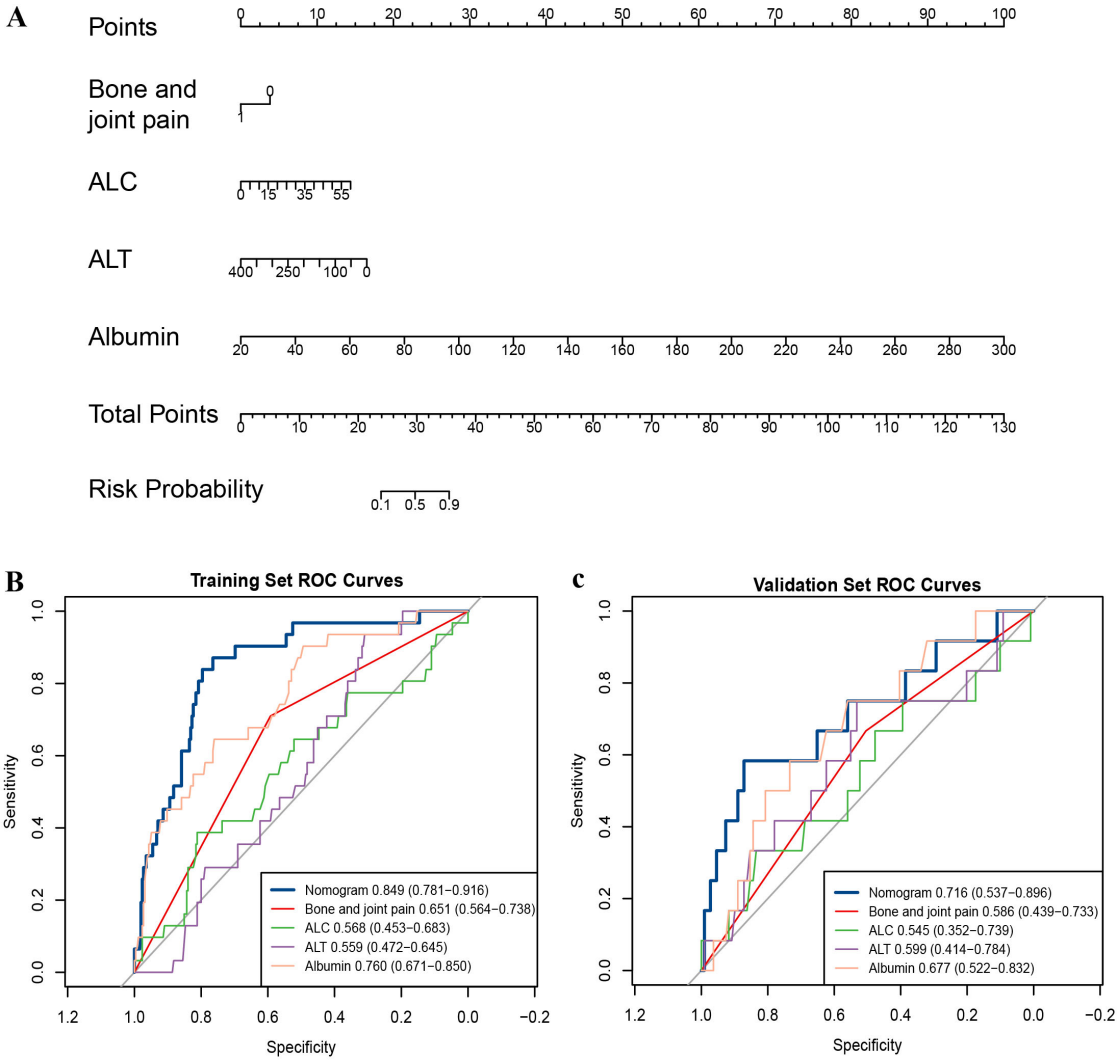
**(A)** Nomogram model. **(B)** ROC curves of the individual predictors and the nomogram model in the training set. **(C)** ROC curves of the individual predictors and the nomogram model in the validation set.

A nomogram for predicting NB risk was developed based on the four independent predictors identified by multivariable logistic regression, including bone and joint pain, ALC, ALT, and albumin (**Figure 2A**). Each predictor was assigned a weighted score, and higher total scores corresponded to a higher probability of NB. The 407 patients were randomly allocated to a training set and a validation set in a 7:3 ratio. The model was established in the training set, and ROC curves for the nomogram and each individual predictor were generated in both the training and validation sets (**Figures 2B, C**). In both datasets, the nomogram achieved a substantially higher AUC than any single predictor, suggesting superior discriminative ability of the combined model over individual clinical or laboratory variables.

### 3.6 Model evaluation

**Figure 3:**
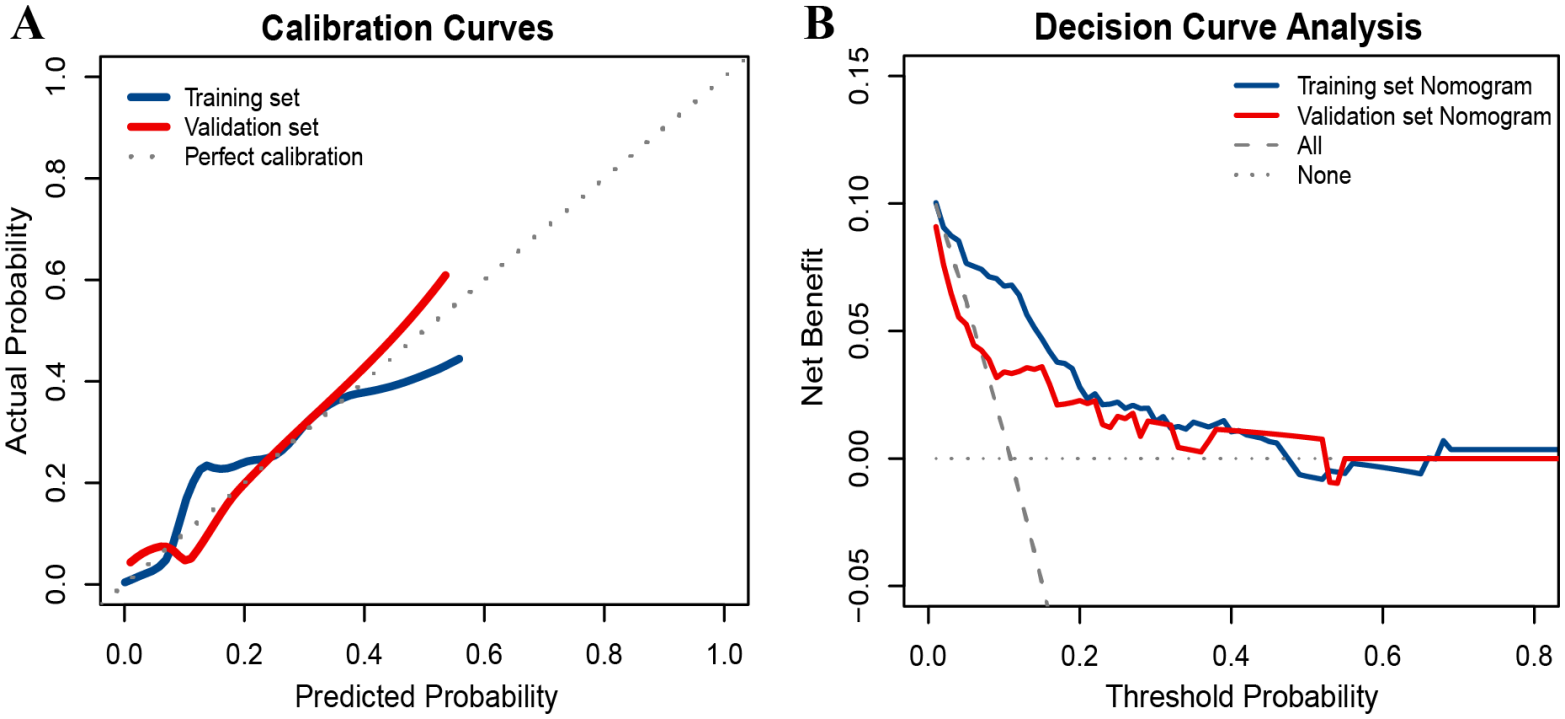
Model evaluation. **(A)** Calibration curves of the nomogram model in the training set and validation set, respectively. **(B)** DCA curves of the nomogram model in the training set and validation set, respectively.

In terms of calibration, the bootstrap calibration curve in the training set showed good agreement between the predicted probabilities and the observed event rates, with the curve lying close to the ideal 45° reference line, indicating satisfactory internal calibration of the model. In the validation set, the LOESS-smoothed calibration curve likewise demonstrated a good fitting trend (**Figure 3A**). The DCA showed that within a certain range of threshold probabilities, approximately corresponding to the risk interval in which clinical intervention might realistically be considered, use of the nomogram to guide clinical decision-making yielded a higher net benefit than either the “intervene in all” or “intervene in none” strategy. The trends of the curves were generally consistent between the training and validation sets (**Figure 3B**). These findings indicate that the proposed NB risk prediction model not only demonstrated good statistical performance, but also showed potential for clinical application.

## 4 Discussion

In this single-center retrospective cohort study, we systematically analyzed 407 hospitalized patients with confirmed brucellosis, among whom 10.6% (43/407) were diagnosed with NB. By comparing the clinical and laboratory characteristics between patients with and without NB, we found that bone and joint pain, ALC, ALT, and albumin levels were closely associated with the occurrence of NB. Based on these factors, we developed a corresponding nomogram to estimate the individualized risk of NB in patients with brucellosis, which may facilitate the early identification and treatment of NB without causing excessive examinations or overtreatment. The occurrence of NB may be closely related to the absence of typical osteoarticular symptoms, less obvious liver function impairment, and a relatively active cellular immune response. In clinical practice, even when laboratory findings appear relatively “normal” in patients with brucellosis, the possibility of NB should still be highly suspected if neurological symptoms are present, so as to avoid missed diagnosis and delayed treatment.

In patients with brucellosis who present with bone and joint pain, the probability of being diagnosed with NB is only 0.23 times that of individuals without this symptom (OR = 0.23), which contrasts with the classic understanding of brucellosis as primarily affecting the bones and joints. One possible explanation is that typical osteoarticular symptoms are more likely to raise clinical suspicion and prompt early medical attention and appropriate treatment, thereby reducing the risk of progression to NB. This also suggests that in a multivariable differential diagnosis model, the presence of “bone and joint pain” alone may point toward other diagnoses.

The elevated ALC observed in patients with NB may mainly be related to the intracellular parasitic nature of Brucella, the predominance of cell-mediated immune responses in the inflammatory process, bacterial inhibition of lymphocyte apoptosis, and the lymphocyte-dominant immune infiltration within the central nervous system (Tolomeo et al., 2003; Tajerian et al., 2024), which may indicate activation of cellular immunity associated with central nervous system involvement. In addition, central nervous system infections are often accompanied by the dynamic redistribution of peripheral immune cells between the blood and cerebrospinal fluid. Previous studies have shown that the proportion of lymphocytes in the cerebrospinal fluid is increased in patients with NB (Bouferraa et al., 2021; Zhang et al., 2025), suggesting that lymphocytes play an important role in the development and persistence of NB. Therefore, an elevated peripheral blood ALC may serve as an immunological marker of NB occurrence rather than merely reflecting the severity of inflammation.

ALT is a sensitive biochemical indicator of hepatocellular injury. Previous studies have suggested that patients with non-NB often present with varying degrees of hepatic involvement, and elevations in ALT and AST are therefore not uncommon (Shrestha et al., 2022; Çakır Kıymaz and Elaldı, 2025; Arslan et al., 2026). In contrast, NB is primarily characterized by central nervous system involvement, and liver injury may be relatively mild in these patients. Thus, lower ALT levels may reflect differences in patterns of organ involvement during Brucella infection. However, this finding may not be solely explained by differences in organ tropism. Patients with NB may seek medical attention earlier because neurological manifestations, such as persistent headache, altered mental status, or meningeal irritation, are often more alarming and prompt earlier hospitalization. As a result, liver enzyme abnormalities may be less pronounced at the time of diagnosis. In addition, NB patients may have fewer underlying liver diseases or milder baseline hepatic involvement, which could also contribute to lower ALT levels. Taken together, these findings suggest that ALT itself may not be a direct risk factor for NB, but rather an indirect marker reflecting differences in disease phenotype, timing of clinical presentation, baseline hepatic status, and patterns of organ involvement among distinct clinical subtypes of brucellosis. In other words, in patients with relatively low ALT levels and less evident hepatic involvement, Brucella may be more likely to invade the central nervous system through hematogenous dissemination or immune-mediated mechanisms, although the exact biological basis underlying this association warrants further investigation.

Albumin is generally regarded as a negative acute-phase reactant, and its level usually decreases in the presence of severe infection or systemic inflammation (Wiedermann, 2021). However, in the present study, elevated albumin levels were associated with the occurrence of NB, which may suggest that the overall inflammatory response in patients with NB is relatively localized or that hepatic synthetic function is relatively preserved. NB often follows a subacute or chronic course, and compared with brucellosis that is commonly accompanied by marked hepatosplenomegaly and hepatocellular injury, its systemic inflammatory burden may be lower (Zhuang et al., 2024). Under these circumstances, albumin levels may not decline markedly and may even appear relatively elevated in some patients. In addition, albumin has biological functions including antioxidative effects, immunomodulation, and maintenance of blood-brain barrier stability. Whether its elevation is related to a compensatory protective mechanism during central nervous system inflammation remains to be further clarified.

The nomogram constructed on the basis of the above four independent factors demonstrated good discrimination and calibration in both the training and validation sets, indicating strong stability and generalizability. Compared with any single clinical symptom or laboratory parameter, a nomogram integrating multiple readily available indicators allows for more refined individual risk stratification and may help clinicians identify patients at high risk of NB at an early stage of hospitalization. Decision curve analysis further showed that, within a reasonable range of threshold probabilities, using this model to guide clinical decision-making provided a greater net benefit than either the “intervene in all” or “intervene in none” strategy. From a clinical application perspective, this model may effectively balance the need for early detection against the risk of overdiagnosis and overtreatment.

This study has several strengths. First, the cases were obtained from a tertiary general hospital in a brucellosis-endemic area, with a relatively large sample size encompassing diverse clinical and laboratory features. Second, strict inclusion and exclusion criteria were used to exclude multiple comorbid conditions that might interfere with the interpretation of neurological manifestations. Third, the model was assessed using a training set and an internal validation set, and its statistical performance and clinical utility were further validated by calibration analysis and DCA. Several limitations of this study should be acknowledged. First, this was a single-center retrospective study and was therefore inevitably subject to potential selection and information biases, which may limit the generalizability of the findings. Second, although detailed clinical, cerebrospinal fluid, neuroimaging, and treatment-related characteristics of patients with neurobrucellosis were collected, the number of NB events was relatively small. Although the final model was parsimonious and the overall events per variable satisfied the recommended requirement for logistic regression analysis, the limited number of events precluded the development of separate prediction models stratified by clinical subtype, cerebrospinal fluid profile, or neuroimaging features. Third, musculoskeletal symptoms may have influenced clinicians’ decision-making regarding lumbar puncture, thereby potentially affecting the diagnosis of NB. Moreover, because comprehensive musculoskeletal imaging was not performed in all patients, occult focal osteoarticular involvement could not be completely ruled out. Therefore, future prospective multicenter studies with larger sample sizes are warranted to further verify the robustness, external generalizability, and clinical applicability of the proposed model, and to further develop subtype-specific prediction models.

In summary, this study developed a nomogram based on readily available clinical indicators to assess the risk of NB in patients with brucellosis, and preliminarily demonstrated its good predictive performance and potential clinical utility. Future multicenter prospective studies conducted across different regions and healthcare settings are needed to externally validate the model and further refine its applicability.

## Data Availability

The raw data supporting the conclusions of this article will be made available by the authors, without undue reservation.
